# Bilateral primary adrenal lymphoma

**DOI:** 10.1002/jgf2.70042

**Published:** 2025-06-26

**Authors:** Rio Shimizu, Shogo Shirota

**Affiliations:** ^1^ Department of General Medicine Osaka Medical and Pharmaceutical University Hospital Osaka Japan

**Keywords:** endocrinology, hematology

A previously healthy 83‐year‐old woman was admitted to our hospital with a three‐week history of fever and a one‐day history of lower back pain. On physical examination, her body temperature was 38.9°C, blood pressure was 100/61 mmHg, and pulse rate was 112 beats/min. Palpable masses in the bilateral costal areas and bilateral costovertebral angle tenderness to percussion were noted. Laboratory tests revealed an elevated white blood cell count of 10,470/μL, lactate dehydrogenase level of 603 U/L, C‐reactive protein of 15.64 mg/dL, and soluble interleukin‐2 receptor of 2403 U/mL. Cortisol level was 4.57 μg/dL, and adrenocorticotropic hormone was 549.0 pg/mL. Adrenocorticotropic hormone stimulation test revealed a peak cortisol level of 4.99 μg/dL. Abdominal contrast‐enhanced computed tomography (CT) demonstrated bilateral adrenal enlargement with heterogeneous enhancement (Figure 1). The right and left adrenal glands were 7 and 6 cm long, respectively. CT‐guided biopsy of the right adrenal mass revealed proliferation of atypical cells with a high nuclear‐to‐cytoplasmic ratio on hematoxylin and eosin staining. Immunohistochemical staining revealed CD20 and Bcl‐6 positivity, consistent with diffuse large B‐cell lymphoma (Figure 2A,B). Based on these findings, the patient was diagnosed with bilateral primary adrenal lymphoma and primary adrenal insufficiency. She was treated with hydrocortisone at a dose of 20–30 mg per day, adjusted according to her clinical condition, and subsequently started on chemotherapy with rituximab, cyclophosphamide, doxorubicin, etoposide, and prednisone, but died on the 117th hospital day.

**FIGURE 1:**
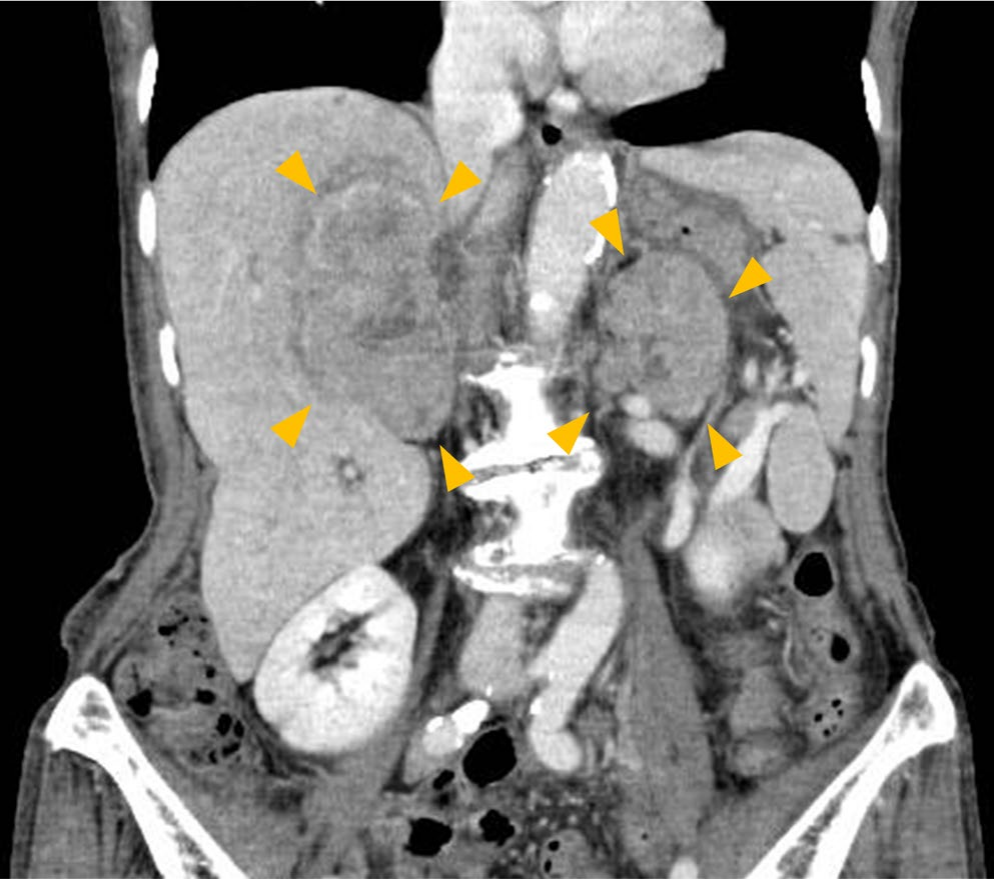
Abdominal contrast‐enhanced CT shows bilateral adrenal enlargement with heterogeneous enhancement.

**FIGURE 2:**
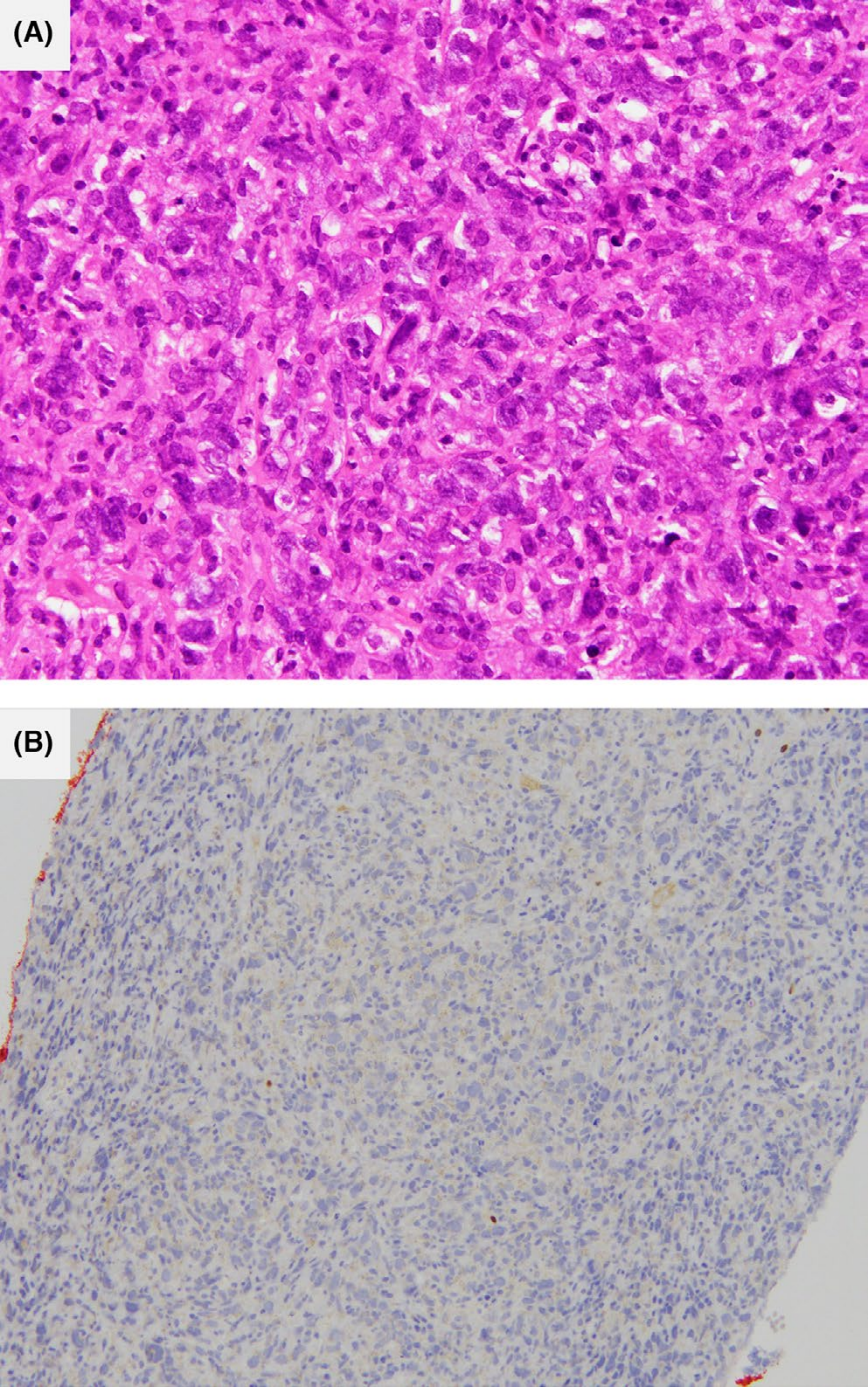
Histological findings of CT‐guided biopsy of the right adrenal mass reveal proliferation of atypical cells with a high nuclear‐to‐cytoplasmic ratio on hematoxylin and eosin staining (A). Immunohistochemical staining shows Bcl‐6 positivity (B).

Primary adrenal lymphoma (PAL) is a rare lymphoma that accounts for approximately 1% of all malignant lymphomas, of which 80% are bilateral.^1^, ^2^ PAL is characterized by its large size (>5 cm) but lacks specific imaging features distinguishing it from other adrenal malignancies.^2^, ^3^ Other differential diagnoses of bilateral adrenal masses include pheochromocytoma, tuberculosis, or metastases. The average size of PAL masses has been reported to be 5.5 cm, while other bilateral adrenal masses are generally smaller, often less than 5 cm.^4^ In addition, in cases of bilateral adrenal masses, adrenal insufficiency has been reported in 57% of PAL and 94% of tuberculosis and is less common in pheochromocytoma and metastasis.^4^ If bilateral adrenal masses >5 cm are found in a patient with fever and back pain, it is important to consider PAL, evaluate adrenal function, and perform biopsy promptly.

## AUTHOR CONTRIBUTIONS

**Rio Shimizu:** Writing – original draft; data curation; investigation. **Shogo Shirota:** Writing – review and editing; conceptualization; supervision; project administration.

## FUNDING INFORMATION

This study did not receive any specific grants from agencies in the public, commercial, or non‐profit sectors.

## CONFLICT OF INTEREST STATEMENT

The authors declare that they have no competing interests.

## ETHICS STATEMENT

Ethics approval statement: Ethical approval was not required for this case report in accordance with the policies of our institution.

Patient consent statement: The patient provided written informed consent before her passing for the publication of her medical data and relevant images.

Clinical trial registration: None.

## PERMISSION TO REPRODUCE MATERIAL FROM OTHER SOURCES

No previously published material was used in this case report.

## INFORMED CONSENT STATEMENT

The patient provided written informed consent before her passing for the publication of her medical data and relevant images.

## Data Availability

No datasets were generated or analyzed during the preparation of this case report.
